# Ultrasound-guided lymph node biopsy sampling to study the immunopathogenesis of rheumatoid arthritis: a well-tolerated valuable research tool

**DOI:** 10.1186/s13075-022-02728-7

**Published:** 2022-02-03

**Authors:** Renée H. Fiechter, Janne W. Bolt, Marleen G. H. van de Sande, Caroline J. Aalbers, Robert B. M. Landewé, Mario Maas, Sander W. Tas, Lisa G. M. van Baarsen

**Affiliations:** 1 Department of Rheumatology and Clinical Immunology, Amsterdam Institute for Infection & Immunity, Amsterdam University Medical Centers, Location Academic Medical Center, University of Amsterdam, Amsterdam, The Netherlands; 2 Department of Experimental Immunology, Amsterdam Institute for Infection & Immunity, Amsterdam University Medical Centers, Location Academic Medical Center, University of Amsterdam, Amsterdam, The Netherlands; 3 Amsterdam Rheumatology and Immunology Center (ARC), EULAR & FOCIS Center of Excellence, Amsterdam UMC, Location Academic Medical Center/University of Amsterdam, Meibergdreef 9, 1105 AZ Amsterdam, The Netherlands; 4 Department of Radiology, Amsterdam University Medical Centers, Location Academic Medical Center, University of Amsterdam, Amsterdam, The Netherlands

**Keywords:** Ultrasound-guided lymph node biopsies, Patient’s perspective, Rheumatoid arthritis, Inflammatory diseases, Translational research

## Abstract

**Background:**

Analyses of lymphoid organs are required to further elucidate the pathogenesis of inflammatory diseases like rheumatoid arthritis (RA). Yet, invasive tissue collection methods are scarcely applied, because they are often considered burdensome, although patients do not always consider invasive methods as a high burden. We aimed to investigate the perspectives of study participants undergoing ultrasound-guided inguinal lymph node (LN) needle biopsy sampling and determine the molecular and cellular quantity and quality of LN biopsies.

**Methods:**

Together with patient research partners, questionnaires were developed to evaluate the motives, expectations, and experiences of participants undergoing a LN biopsy. Healthy controls and RA(-risk) patients were asked to complete these questionnaires before and after the procedure. RNA and lymphocyte yields from obtained LN biopsies were also calculated.

**Results:**

We included 50 individuals, of which 43 (86%) reported their pre- and post-procedure experiences. The median reported pain on a 5-point Likert scale (1 not to 5 very painful) was 1. Interestingly, almost all (*n* = 32; 74%) study participants would undergo a second procedure and more than half (*n* = 23; 54%) would encourage others to take part in the LN biopsy study. Motives for current and future participation were mostly altruistic. Inguinal hematoma occurred frequently, but no other significant or unexpected complications ensued. The LN biopsies yielded sufficient and high-quality RNA and lymphocyte numbers.

**Conclusions:**

Ultrasound-guided inguinal LN biopsy sampling is well-tolerated, safe, and provides sufficient material for further molecular and cellular analyses. Our participants’ positive experiences endorse the application of this research tool to further elucidate the pathogenesis of RA and other inflammatory diseases.

**Supplementary Information:**

The online version contains supplementary material available at 10.1186/s13075-022-02728-7.

## Background

Rheumatoid arthritis (RA) is a chronic inflammatory disease that is still incurable and requires life-long immunosuppressive treatment [1]. If uncontrolled, RA will result in a lower quality of life due to disability, pain, and other comorbidities [2]. Although multiple treatment options exist, prevention of RA in the preclinical phase of the disease (pre-RA) would be desirable and is the ultimate goal of an increasing number of research groups, including our own [3, 4].

Studying the immune system in more detail through ultrasound-guided lymph node (LN) biopsy sampling could expedite reaching this goal and potentially improve the future of (pre-)RA patients for multiple reasons. LNs not only serve as focal points for initiating immune responses, but also ensure peripheral tolerance [5]. Loss of peripheral tolerance is associated with the development of immune-mediated inflammatory diseases (IMIDs), like RA, due to the occurrence of activated auto-reactive immune cells and auto-antibodies such as rheumatoid factor (RF) and anti-cyclic citrullinated protein antibodies (ACPA) [6]. These auto-antibodies can already be present years before the onset of RA [7] in the so-called RA-risk individuals [6, 8, 9]. The at-risk phase is defined as individuals with positive serology for RF and/or ACPA combined with a positive family history for RA or arthralgia [3]. First-degree relatives of RA patients have an especially high risk of developing RA and can experience symptoms such as symmetrical and small joint pain [10]. As systemic autoimmunity precedes synovial inflammation [11] and animal studies [12, 13] have suggested that changes in the LNs may precede those in the synovial tissue, it is important to study LN tissues in more detail. Indeed, we previously discovered through ultrasound-guided LN biopsy sampling that, compared to healthy controls (HCs), multiple LN alterations can be found in RA-risk individuals [7, 14–16], which highlights the potential of this technique to study the pre-clinical or “at-risk” phase of RA. We have also established that LNs of RA patients differ from HCs and/or RA-risk individuals [7, 15, 16]. Moreover, we previously showed that rituximab treatment altered frequencies of immune in inguinal LNs of RA patients, reflecting incomplete B cell depletion with the persistence of switched memory B cells [17]. Ultrasound-guided LN biopsy sampling thus allows for detailed cellular and molecular studies of the immune system beyond the peripheral blood compartment at all disease stages. This may further elucidate RA pathogenesis and identify novel targets: prognostic and/or therapeutic biomarkers, which could be involved in clinical decision-making for personalized treatment, biomarkers that may identify persons that will develop RA or possibly other IMIDs, and finally biomarkers that may be targeted to prevent disease. The latter two are very important in the quest towards disease prevention [18].

Ultrasound-guided LN biopsy sampling is an established diagnostic and research tool in hematology and oncology [19]. However, we noticed that many IMID researchers regard LN biopsy sampling as invasive and burdensome for patients and therefore find it ethically challenging to apply this as a research method. Importantly, patients who undergo invasive procedures do not always consider them a high burden, even when directly compared with non-invasive procedures such as magnetic resonance imaging (MRI) [20]. We previously demonstrated that LN biopsy sampling in (at-risk) patients with RA for research purposes is safe [21]. Nevertheless, LN biopsy sampling is only sporadically applied to study IMIDs.

To validate that ultrasound-guided LN biopsy sampling can be applied safely in IMID research studies, we investigated the experiences of HCs, individuals at risk of developing RA, and RA patients undergoing the procedure. In addition, we compared the healthy controls’, RA-risk individuals’, and patients’ perspectives and intentions of undergoing ultrasound-guided LN biopsy sampling. Finally, we determined the cellular and molecular yields and quality of the obtained LN tissues that have been collected in our biobank over the years to further demonstrate the research potential of this method.

## Methods

We set up this observational study to investigate the perspectives of study participants undergoing ultrasound-guided inguinal LN needle biopsy sampling. In addition, we determined molecular and cellular quantity and quality of LN biopsies from our biobank to highlight the scientific value of this research tool.

### Patient involvement and questionnaire development

Together with patient research partners and researchers within the European research consortium Euro-TEAM (FP7 EU funded research project 305549), we developed two questionnaires: the first explored the participants’ feelings, understandings, and motives before the LN biopsy procedure; the second examined their experiences, complications, and future intentions after the procedure (see Additional files 1 and 2) [22]. Patient research partners helped to draft the questions, including how to assess the burden of the intervention, the manner of response, and advised on study design.

### Study participants

Participants consisted of HCs, RA patients, and RA-risk individuals. RA patients and RA-risk individuals were recruited by our study team after a referral from their own health care professional in our outpatient clinic. RA-risk individuals had increased serum IgM RF and/or ACPA levels and were either suffering from arthralgia or had a first-degree relative with RA. HCs were recruited via other participants or via flyers and/or posters in our hospital. All participants were informed by our study team about the aim, procedure, and possible complications. All participants gave written informed consent before the procedure as approved by the Medical Ethics Committee of the Academic Medical Center, Amsterdam, the Netherlands. The participants received travel and parking reimbursement.

### Lymph node biopsy procedure

All participants were asked to complete the first questionnaire before undergoing the LN biopsy sampling procedure, which has been described previously [21]. In short, using ultrasound the radiologist selected one LN in the participants’ groin (often of normal size). Subsequently, the area was disinfected and anesthetized, and multiple 16-gauge needle biopsies were taken. To minimize hematoma formation after the procedure, manual pressure was applied to the area and the participant remained in a resting position for about 30 min. Subsequently, participants received a second questionnaire in duplicate and were asked to complete the questionnaires at day 1 and day 5 after the procedure and to return them by mail. Additionally, we checked up on our study participants 5 days after the procedure by telephone and reminded all study participants to return the questionnaires.

### Lymph node tissue processing

To highlight the scientific value of this research tool, we also calculated RNA and lymphocyte yields of LN biopsy samples from our biobank processed as described previously [14, 16, 17]. Briefly, immediately after collection, biopsies were snap-frozen en bloc in Tissue-Tek OCT compound (Miles, Elkhart, IN, USA) for immunohistochemistry analysis or snap-frozen for RNA isolation. For cellular analyses, LN tissue was placed in medium and passed through a 70-μm (BD Falcon, San Jose, CA) cell strainer as quickly as possible to obtain a single-cell suspension.

### Statistical analysis

Data was analyzed in SPSS Statistics 26, using descriptive statistics (presented as percentages of the total, mean and standard deviation (SD), or median and interquartile ranges (IQR)), and Fisher’s exact test for the differences in pain between HCs, RA-risk, and RA participants. Figures were generated in GraphPad Prism 8.3.0.

## Results

### Patient demographics

Fifty participants were recruited and completed the questionnaire before the biopsy procedure: 34 RA patients, 8 RA-risk individuals, and 8 HCs. The mean age was 58 years ± 13 SD, and 22 (44%) were males. The RA patients had a median disease duration of 5 years (IQR 0–9 years) and low disease activity in general with a median Disease Activity Score-28 (DAS-28) of 2.63 (IQR 2.03–3.83). Eleven RA patients (32%) had moderate disease activity with a DAS-28 > 3.2. Four RA patients did not use any kind of medication for their disease. Of the 30 RA patients that used anti-inflammatory drugs, 14 used conventional disease-modifying antirheumatic drugs (cDMARDs), 1 used cDMARDs in combination with non-steroidal anti-inflammatory drugs (NSAIDs) and corticosteroids, 6 used cDMARDs in combination with NSAIDs, 4 used cDMARDs in combination with corticosteroids, 1 used NSAIDs and corticosteroids, and 4 used NSAIDs only. The response rate for the questionnaires 1 day after the procedure was 86% as 43 participants returned their questionnaires after the biopsy procedure: 29 RA patients, 7 RA-risk individuals, and 7 HCs. The response rate was lower for the questionnaires at day 5 as 34 participants fulfilled these questionnaires.

### Motives, expectations, and understanding of the procedure

**Fig. 1:**
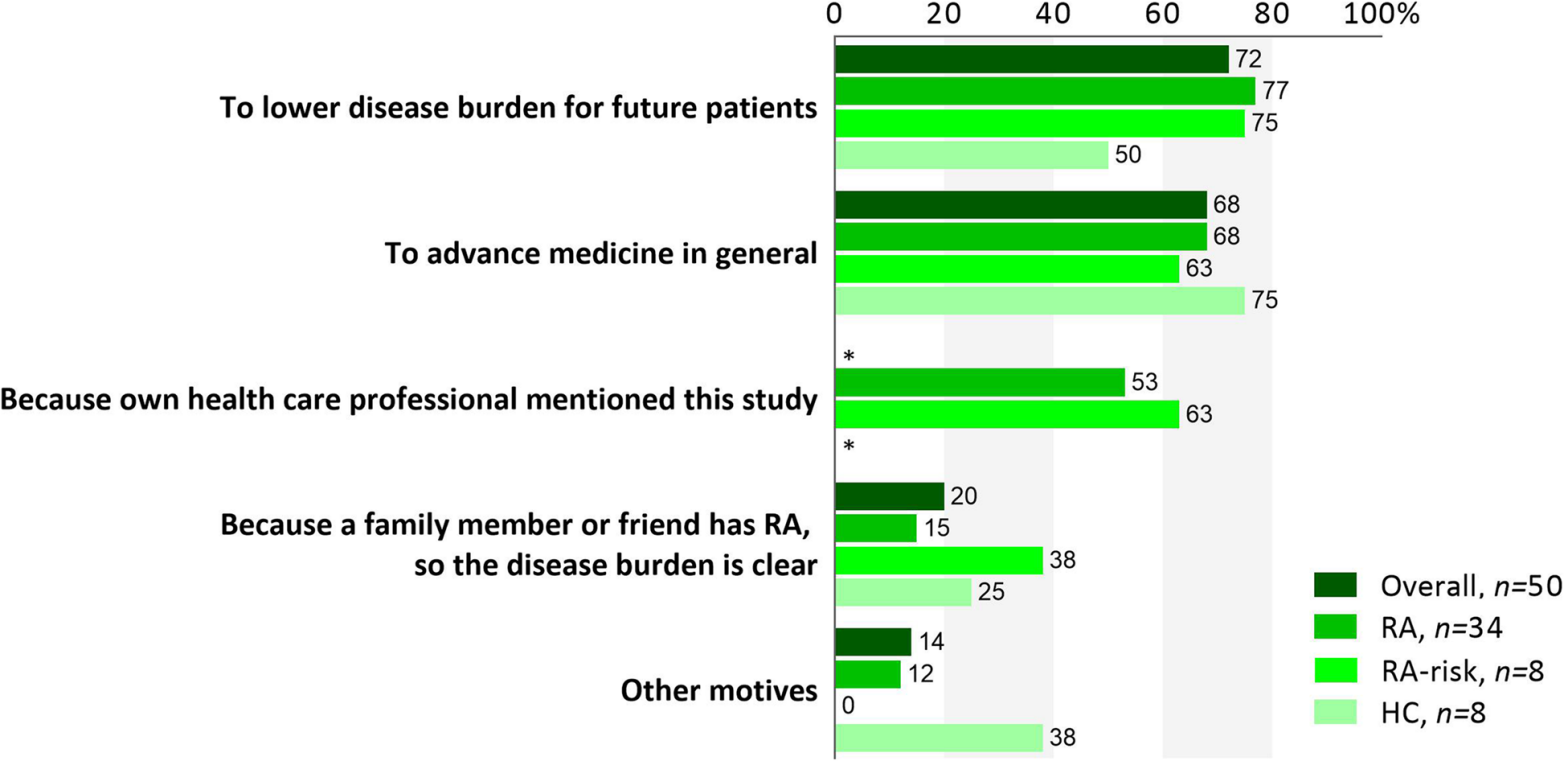
Altruism and scientific advancement as major motives. Participants reported their motivation to participate by choosing from four fixed motives and/or an open option called “other motives.” Most participants were motivated either by altruism or scientific advancement, while more than half of the RA patients and RA-risk individuals reported that the positive attitude of their own health care professional towards this study was one of the reasons for them to partake. Detailed explanations of “other motives” can be found in Additional file 3. Overall includes RA, RA-risk, and HC individuals. RA, rheumatoid arthritis patients; RA-risk, individuals at risk for developing RA; HC, healthy controls. *Not applicable

Participants could choose from one or more of four fixed motives to participate and/or an open option called “other motives.” Most participants reported altruism and scientific advancement as prime motives to participate in the study (Fig. 1). Seven (14%) participants had other motives of which three mentioned they participated hoping it would advance disease management and would relieve their own symptoms in the future (see Additional file 3). More than half of the RA patients (*n* = 18; 53%) and RA-risk individuals (*n* = 5; 63%) reported that they were extra motivated to participate since their own health care professional expressed a positive attitude towards this study, complementary to the information provided by the study team. HCs were not recruited through health care professionals and accordingly did not choose this option. Overall, our participants did not feel anxious nor did they dread the procedure (Table 1). Moreover, they felt well-informed about the different aspects of the procedure (Table 1).

**Table 1:** Perceptions and understandings towards ultrasound-guided lymph node biopsy sampling before the procedure

	Overall, ***n*** = 50	RA, ***n*** = 34	RA-risk, ***n*** = 8	HC, ***n*** = 8
Anxiousness	16 [3–44]	13 [3–48]	20 [3–47]	16 [2–20]
Dreading the procedure	15 [3–48]	14 [3–42]	19 [1–72]	17 [2–44]
Understanding of goal and background	92 [83–98]	94 [87–99]	86 [78–91]	84 [70–99]
Understanding of what to expect during the procedure	89 [76–97]	91 [74–98]	85 [79–90]	83 [71–97]
Understanding of possible complications	90 [83–98]	94 [84–99]	86 [85–99]	83 [70–91]
Understanding of aftercare	90 [79–98]	92 [77–99]	86 [82–92]	89 [70–91]

The questions were scored on a visual analog scale of 0–100. The median and interquartile ranges are shown

*RA* rheumatoid arthritis patients, *RA-risk* individuals at risk for developing RA, *HC* healthy controls

### Post-biopsy experiences

**Fig. 2:**
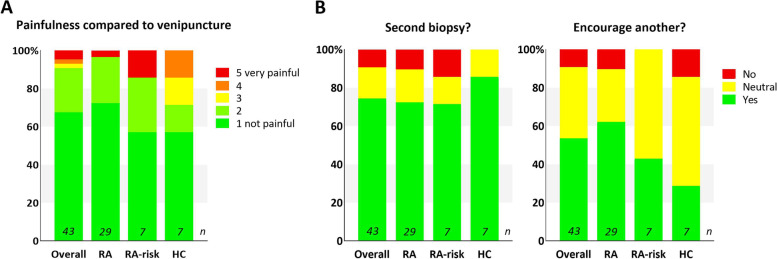
Low painfulness and positive future intentions. **A** Study participants scored the pain they experienced during the ultrasound-guided inguinal lymph node biopsy on a 5-point Likert scale from 1 “not painful” to 5 “very painful” compared to venipuncture. The majority (91%) of responders scored either 1 or 2. Pain scores between the various groups did not differ by Fisher’s exact test. **B** Study participants reported their willingness to undergo a second biopsy and to encourage someone else to participate in a similar study by choosing yes, no, or neutral. The majority of all responders were willing to undergo a second biopsy (74%) and around half of the participants were willing to encourage someone else (54%), while around half of RA-risk individuals and healthy controls were neutral about encouraging someone else (54%). Overall includes RA, RA-risk, and HC individuals. RA, rheumatoid arthritis patients; RA-risk, individuals at risk for developing RA; HC, healthy controls

As the results of the questionnaires of days 1 and 5 after the biopsy closely aligned for each individual patient, we only show data from day 1 here (see Additional files 4 and 5 for data of day 5). Our participants scored the painfulness of the procedure compared to venipuncture on a 5-point Likert scale from 1 “not painful” to 5 “very painful,” as shown in Fig. 2A. Thirty-nine (91%) of all 43 responders scored either 1 or 2 and thus experienced no or a little pain during the LN biopsy procedure. Fisher’s exact showed no differences in pain between the various groups of participants. In a multiple-choice question, 16 (37%) responders self-reported minor complications, mostly hematomas (*n* = 15, 94%). Five patients observed some wound leakage, three patients experienced severe pain after the procedure, and one patient observed some bleeding of the wound. None of the patients experiencing these self-reported complications contacted us to report these complications. Also, during our check-up by telephone 5 days after the procedure, we asked participants about the possible complications, and except for hematomas, no complications were mentioned. None reported infections. The majority of responders (*n* = 40; 93%) indicated that the procedure was well-explained; only three (7%) responders recommended a better explanation of either the biopsy procedure, the local anesthesia, or possible complications.

Participants were asked if they would consider a second biopsy and if they would encourage another person to participate in a study involving a LN biopsy (Fig. 2B) and to explain their choice (see Additional file 6). Thirty-two (74%) of all responders would consider undergoing a second biopsy. Most participants clarified this choice by the procedure not being painful and by the importance of scientific advancement—“I barely felt it and it is for a very good cause.” More than half of all subjects (*n* = 23; 54%) would encourage another person to participate in a study with a LN biopsy, but more than half of the RA-risks (*n* = 4; 57%) and HCs (*n* = 4; 57%) were neutral. Participants mostly explained their decision by not knowing anyone to recommend or because they valued autonomous decisions—“I would describe my experiences, but would leave the decision up to that person.”

### Acceptance rate

We aimed to evaluate motives and characteristics from individuals who did not want to participate in order to assess the general acceptance rate of the LN biopsy; however, these individuals were not inclined to fill in such a questionnaire. To evaluate if disease activity affects the acceptance rate in RA patients, we compared the results in RA patients with low (DAS-28 < 3.2) versus moderate disease activity (DAS-28 > 3.2). We observed no difference in pain experienced during the biopsy between the 19 patients with low (DAS-28 < 3.2) and 10 patients with moderate (DAS-28 > 3.2) disease activity as respectively 18/19 and 10/10 patients scored either a 1 or 2 for painfulness compared to venipuncture. Similarly, percentages of patients who were willing to undergo a second procedure were comparable in the low and moderate disease activity groups (14/19 versus 7/10, respectively). On the contrary, 9/10 patients in the moderate disease activity group were willing to encourage someone else to participate in a LN biopsy study compared to 9/19 in the low disease activity group. Thus, while keeping in mind our limited sample size, higher disease activity does not seem to influence the acceptance rate in our cohort in reference to the RA patients themselves but may affect their willingness to encourage someone else to participate.

### Lymph node biopsy quality

In the current and previous studies, 1–2 LN biopsies were collected from each study participant for RNA isolation. On average, this resulted in good RNA quality and yield in LN samples from 236 participants stored in the LN biobank: median 4.3 μg (IQR 1.6–8.0 μg), which was well-suited for downstream RNA based analyses such as quantitative polymerase chain reaction and genome-wide expression profiling studies. No clear differences were observed between the different diagnoses: 44 RA patients (median 3.9, IQR 2.7–8.2 μg), 135 RA-risk individuals (median 3.9, IQR 1.0–7.8 μg), 25 HCs (median 4.2, IQR 0.9–7.8 μg), and 32 individuals with other types of IMIDs (median 5.4, IQR 3.2–9.4 μg). For cellular studies, we collected lymphocytes from preferably 4 biopsies per participant. The resulting total number of lymphocytes was highly variable between donors: median 1.2 million (IQR 0.4–2.4 million) cells for all 42 participants. Specified per diagnosis, the total number of lymphocytes was for 36 RA patients, a median of 1.3 (IQR 0.6–2.0); for 2 RA-risk individuals, a median of 3.1 (IQR 2.7–3.5); and for 4 HCs, a median of 1.2 (IQR 0.3–2.4) million cells. From four RA patients, only 2 or 3 biopsies were available. More RA-risk individuals should be included before conclusions can be drawn about their higher lymphocyte yield. In general, obtained lymphocytes could be successfully phenotyped using flow cytometry-based technology as well as in vitro expanded [7, 14, 16, 23]. In an earlier cohort study using ultrasound-guided LN biopsies [17], we evaluated tissue quality using immunohistochemistry on tissue sections of 142 study participants. From these tissue sections, we only excluded 8 donors (6%) due to low tissue quality or cutting artefacts, thus confirming an overall good quality of collected LN biopsies. In our experience, the LN biopsy procedure renders sufficient amounts of good-quality tissue to perform downstream state-of-the-art analyses, including (sc)RNA sequencing and immunofluorescent staining methods [7, 15].

## Discussion

In this study, we report that ultrasound-guided LN biopsy sampling is generally well-tolerated by RA patients, RA-risk individuals, and healthy controls, as they experienced no to little discomfort and would be willing to participate again if requested.

Similar to our results, the majority of early arthritis patients undergoing ultrasound-guided synovial biopsies for research purposes experienced no or only minor discomfort and would be willing to repeat the procedure [24]. Many other studies indicated that patients are willing to undergo invasive procedures for research purposes [25–29]. Also, in the context of predictive testing, patients are willing to endure some discomfort for scientific advancement [30]. One of our study participants said it very clearly: “The value for future patients outweighs the little pain and discomfort I endured.” More than half of the participants would also encourage others to participate in a study with repeated biopsies. Interestingly, as known from the literature [31], this percentage increased for patients with more symptoms, since almost all RA patients with a DAS-28 > 3.2 were willing to encourage someone else to participate in a study with repeated biopsies.

This paper shows that RA patients, RA-risk individuals, and HCs can be motivated to participate in a study collecting ultrasound-guided LN biopsies. Participants from all groups were mostly stimulated by altruistic motives: both before the procedure when asked why they would participate in the biopsy study and after the procedure as explanation for their willingness to undergo a second biopsy. Based on the literature, participation in tissue sampling studies for research purposes mostly relies on altruistic or financial motivation since compared to diagnostics, genetic studies, or clinical trials, there are no direct personal benefits [32]. The motives of our study participants align with these studies and the information provided to them (i.e., that this study fostered fundamental research and even if the study would eventually lead to improvement of disease management, this would likely be for future generations). Though travel and parking costs were reimbursed, we did not give additional financial compensation in our studies for ethical reasons.

Patients and researchers are generally less willing to accept procedural risks for research than for diagnostic purposes [26]. Since we observed only minor discomfort and no unexpected or major complications, our results reinforce our previous paper stating that this procedure is safe to apply in research and might potentially be of use in preventive, diagnostic, prognostic, or even therapy-predictive settings in the future [21].

Of all secondary lymphoid organs in the body, lymph nodes seem most reflective of systemic immunity while also being relatively easily accessible for image-guided sampling in the outpatient clinic. Our center prefers the 16 G × 13 cm Bard Magnum disposable core tissue biopsy needle, but other techniques are known to obtain similar tissue yields [19]. Since in our obtained biopsies, RNA, and cell yields were sufficient both in numbers and in quality for all planned experiments to be conducted, we regard this procedure as a high-potential and valuable research tool [7, 14–16].

As for the limitations, since the individuals who did not want to participate in our study were not inclined to complete a questionnaire about their reasons for not participating, our study contains a selection bias. Therefore, it is difficult to assess, e.g., whether the acceptance rate in RA patients relies on disease activity. The addition of questionnaires to uncover why people were unwilling to participate would be advised for future comparable studies. We observed a lower response rate on day 5 compared to day 1. Nevertheless, we do not think that this influences our outcomes as the results of days 1 and 5 were mostly similar when linked to the individual participant. Patients that did not complete the questionnaire at day 5 most often had no complaints at day 1. Despite these limitations, this study’s primary strength is the incorporation of the patient’s perspective already in the study design, as patient research partners helped to develop the questionnaires. Another strength is the combination of quantitative and qualitative questions, providing both objective measurements and participants’ own opinions.

Willingness of patients to participate in translational research projects is essential to advance medicine, because only patient samples can give a true insight into the complex pathophysiology of human disease. As loss of peripheral tolerance in secondary lymphoid organs contributes to autoimmunity, studying LN tissue may help elucidate RA onset, yield novel diagnostic markers, and perhaps even therapy-predictive biomarkers for RA-risk individuals, which may allow early diagnosis and treatment during the (preventive) “window of opportunity.” Eventually, this may lead to the development of drugs that treat autoimmunity rather than inflammation [1]. Consequently, supported by the positive experiences from our participants, we advocate wide application of this well-tolerated research tool to advance human LN studies in IMIDs and to reach these goals.

## Conclusions

Ultrasound-guided inguinal LN biopsy sampling is well-tolerated, safe, and provides sufficient material for further molecular and cellular analyses. Our participants’ positive experiences endorse the application of this research tool to further elucidate the pathogenesis of RA and other inflammatory diseases.

## Supplementary Information

**Additional file 1.** Questionnaire before ultrasound-guided lymph node biopsy (in Dutch).

**Additional file 2.** Questionnaire 1 and 5 days after ultrasound-guided lymph node biopsy (in Dutch).

**Additional file 3.** Explanatory statements mentioned when motives before biopsy were noted as “other”.

**Additional file 4.** Results of day 5.

**Additional file 5.** Self-reported complications at day 5.

**Additional file 6.** Explanatory statements that participants mentioned for their choice of yes/neutral/no considering a second ultrasound-guided lymph node biopsy or encouraging another to do so.

**Additional file 7.** Questionnaire before ultrasound-guided lymph node biopsy (in English).

**Additional file 8.** Questionnaire 1 and 5 days after ultrasound-guided lymph node biopsy (in English).

## Data Availability

The datasets used and/or analyzed during the current study are available from the corresponding authors on reasonable request.

## Abbreviations

ACPA: Anti-cyclic citrullinated protein antibodies
cDMARDs: Conventional disease-modifying antirheumatic drugs
DAS-28: Disease Activity Score - 28
HCs: Healthy controls
IMIDs: Immune-mediated inflammatory diseases
IQR: Interquartile range
LN: Lymph node
NSAIDs: Non-steroidal anti-inflammatory drugs
Pre-RA: Preclinical phase of rheumatoid arthritis
RA: Rheumatoid arthritis
RA-risk: At risk of developing rheumatoid arthritis
RF: Rheumatoid factor
SD: Standard deviation
