# Supplementary material for: Ultrasound-guided lymph node biopsy sampling to study the immunopathogenesis of rheumatoid arthritis: a well-tolerated valuable research tool
Source: Arthritis Res Ther. 2022 Feb 3;24:36. doi: 10.1186/s13075-022-02728-7 (PMC8812012; doi:10.1186/s13075-022-02728-7)
Supplement: Supplementary file 1 — Additional file 1. Questionnaire before ultrasound-guided lymph node biopsy (in Dutch). [file 13075_2022_2728_MOESM1_ESM.pdf]

Additional file 1: Questionnaire before ultrasound-guided lymph node biopsy (in Dutch)

Vraag 1. Geeft u aub uw geslacht aan:

Man	☐
Vrouw	☐

Vraag 2. Geeft u aub uw leeftijd aan:

………………. jaar

De volgende vragen gaan over uw ervaringen in het verleden met betrekking tot het ondergaan van een biopsie of een andere medische procedure.

Vraag 3.

a. Heeft u ooit eerder een lymfeklierbiopsie onder plaatselijke verdoving ondergaan?

Nee	☐	Als u 'nee' heeft geantwoord: ga naar vraag 4 aub
Ja	☐	

b. Als u vraag 3a met 'Ja' heeft beantwoord: hoe vaak heeft u eerder een lymfeklierbiopsie onder plaatselijke verdoving ondergaan?

………………. keer

c. Als u vraag 3a met 'Ja' heeft beantwoord: wanneer was de laatste keer dat u een lymfeklierbiopsie onder plaatselijke verdoving heeft ondergaan?

………/………. (maand/jaar)

Vraag 4.

a. Heeft u ooit eerder een procedure onder plaatselijke verdoving ondergaan?

Nee	☐	Als u ‘nee’ heeft geantwoord: ga naar vraag 5 aub
Ja	☐	

b. Als u vraag 4a met ‘Ja’ heeft beantwoord: hoe vaak heeft u eerder een procedure onder plaatselijke verdoving ondergaan?

………………. keer

c. Als u vraag 4a met ‘Ja’ heeft beantwoord: wanneer was de laatste keer dat u een procedure onder plaatselijke verdoving heeft ondergaan?

………/………. (maand/jaar)

Vraag 5. Waarom heeft u besloten deel te nemen aan de studie? Geef hieronder de reden(en) aan door middel van een kruisje. U kunt meerdere redenen aankruisen.

U heeft een familielid/vriend met reumatoïde artritis (RA) en weet daarom hoe RA iemands leven kan beïnvloeden en wil daarom helpen	☐
U wilt helpen om de ziektelast van RA voor toekomstige patiënten te verminderen	☐
U gelooft dat het belangrijk is om, wanneer mogelijk, deel te nemen aan onderzoeksprojecten om de geneeskunde te bevorderen	☐
U heeft over het onderzoek gehoord van uw huisarts/reumatoloog en wil graag helpen	☐
Anders, namelijk………………………………………………………………………………………<br>………<br>………<br>………<br>………	☐

Vraag 6.
Geeft u aub op onderstaande lijn door middel van een verticaal streepje aan hoe goed u zich voorbereid vindt om de lymfeklierbiopsie te ondergaan met betrekking tot:

a. het begrijpen van het doel van de procedure en de achtergrondinformatie

____

0 mm *100 mm*

Volledig niet begrepen Volledig begrepen

b. wat te verwachten tijdens de procedure

____

0 mm *100 mm*

Volledig niet begrepen Volledig begrepen

c. nazorg

____

0 mm *100 mm*

Volledig niet begrepen Volledig begrepen

d. mogelijke complicaties

____

0 mm *100 mm*

Volledig niet begrepen Volledig begrepen

Vraag 7.
Geeft u aub op onderstaande lijn door middel van een verticaal streepje aan hoe angstig u bent om de biopsie te ondergaan:

____

0 mm *100 mm*

Ik ben niet angstig Ik ben zeer angstig

Vraag 8.
Geeft u aub op onderstaande lijn door middel van een verticaal streepje aan in welke mate u tegen het ondergaan van de biopsie opziet:

____

0 mm *100 mm*

Ik zie niet op tegen de procedure Ik zie erg op tegen de procedure

Vraag 9.
Geeft u aub hieronder door middel van een kruisje aan of u met de volgende medicatie moest staken in verband met het ondergaan van de biopsie:

Bloedverdunners	☐
Pijnmedicatie	☐

Heeft u opmerkingen over deze vragenlijst? Geeft u dan graag hieronder een toelichting.

....
....
....
....
....
....
....
....
....
....
....
....
....
....

Hartelijk dank voor uw medewerking!
