# Supplementary material for: Ultrasound-guided lymph node biopsy sampling to study the immunopathogenesis of rheumatoid arthritis: a well-tolerated valuable research tool
Source: Arthritis Res Ther. 2022 Feb 3;24:36. doi: 10.1186/s13075-022-02728-7 (PMC8812012; doi:10.1186/s13075-022-02728-7)
Supplement: Supplementary file 2 — Additional file 2. Questionnaire 1 and 5 days after ultrasound-guided lymph node biopsy (in Dutch). [file 13075_2022_2728_MOESM2_ESM.pdf]

Additional file 2: Questionnaire 1 and 5 days after ultrasound-guided lymph node biopsy (in Dutch)

Vraag 1.

Vergeleken met bloed prikken is de lymfeklierbiopsie (geef hieronder door middel van een kruisje aan wat voor u van toepassing is):

Niet pijnlijk Zeer pijnlijk

☐	☐	☐	☐	☐
1	2	3	4	5

Vraag 2.

a. Heeft u complicaties ervaren na afloop van de lymfeklierbiopsie?

Nee	☐	Als u 'nee' heeft geantwoord: ga naar vraag 3 aub
Ja	☐	

b. Als u vraag 2a met 'Ja' heeft beantwoord: geeft u aub door middel van een kruisje aan welke complicatie(s) u heeft ervaren:

1. Bloeduitstorting:

Nee	☐
Ja	☐

2. Lekkage van het wondje:

Nee	☐
Ja	☐

3. Bloeding:

Nee	☐
Ja	☐

4. Ontsteking:

Nee	☐
Ja	☐

5. Hevige pijn:

Nee	☐
Ja	☐

6. Andere complicatie die niet hierboven vermeld staat:

Nee	☐
Ja, namelijk………………………………………………………………………………………………… ……	☐

Vraag 3.

a. Welke aspecten van de lymfeklierbiopsie hadden achteraf gezien beter kunnen worden toegelicht/uitgelegd?

Geen, ik ben tevreden met de gegeven informatie	☐

óf:

Meer gedetailleerde uitleg over de procedure zelf	☐
De verdovingsprocedure	☐
Nazorg	☐
Te verwachten complicaties	☐
Anders, namelijk ………………………………………………………………………………………… ……… ………	☐

b. Als u een van deze opties heeft aangevinkt, beschrijft u aub welke informatie zal helpen om beter voorbereid te zijn op de lymfeklierbiopsie:

………
………
………
………
………

Vraag 4.

Zijn er achteraf gezien aanvullingen waarmee u denkt dat de lymfeklierbiopsie beter te verdragen zou zijn geweest?

Geen aanvullingen	☐
Iemand die stap voor stap tijdens de lymfeklierbiopsie uitlegt wat er gebeurt	☐
Afleiding tijdens de procedure, bijvoorbeeld door middel van naar muziek of leuke grappen te luisteren	☐
Anders, namelijk………………………………………………………………………………………… …… ……	☐

Vraag 5.

a. Zou u overwegen om nog een keer een lymfeklierbiopsie te ondergaan?

Nee	☐
Ja	☐
Neutraal	☐

b. Geeft u aub commentaar op uw keuze:

……<br>……<br>……	☐

Vraag 6.

a. Zou u iemand anders aansporen om mee te doen aan een studie waarbij lymfeklierbiopten van een lymfeklier in de lies worden genomen?

Nee	☐
Ja	☐
Neutraal	☐

b. Geeft u aub commentaar op uw keuze:

……<br>……<br>……	☐

Vraag 7.

Zou u het op prijs stellen wanneer u op de hoogte wordt gebracht over de studie en de studieresultaten nadat de studie is afgerond?

Nee	☐
Ja	☐

Heeft u opmerkingen over deze vragenlijst? Geeft u dan graag hieronder een toelichting.

……
……
……
……
……
……

Voor deze vragenlijsten voor en na de ingreep:

Indien van toepassing, zou u willen aangeven welke vragen voor u moeilijk te beantwoorden waren of niet goed te begrijpen waren (onduidelijk)?

Zou u hierbij willen aangeven hoe de betreffende vraag volgens u beter gesteld kan worden?

………
………
………
………
………
………
………
………
………
………

Hartelijk dank voor uw medewerking!
