# Supplementary material for: Ultrasound-guided lymph node biopsy sampling to study the immunopathogenesis of rheumatoid arthritis: a well-tolerated valuable research tool
Source: Arthritis Res Ther. 2022 Feb 3;24:36. doi: 10.1186/s13075-022-02728-7 (PMC8812012; doi:10.1186/s13075-022-02728-7)
Supplement: Supplementary file 3 — Additional file 3. Explanatory statements mentioned when motives before biopsy were noted as “other”. [file 13075_2022_2728_MOESM3_ESM.pdf]

Additional file 3: Explanatory statements mentioned when motives before biopsy were noted as "other"

Other explanatory statements for motives before biopsy
My son-in-law motivated me / My son-in-law participates in the study
I have rheumatoid arthritis myself
To solve my own symptoms
Because I was asked
I hope RA can be cured in the future and I will not be hindered anymore by this disease
For study purposes
Because participating in research is important
