# Supplementary material for: Ultrasound-guided lymph node biopsy sampling to study the immunopathogenesis of rheumatoid arthritis: a well-tolerated valuable research tool
Source: Arthritis Res Ther. 2022 Feb 3;24:36. doi: 10.1186/s13075-022-02728-7 (PMC8812012; doi:10.1186/s13075-022-02728-7)
Supplement: Supplementary file 4 — Additional file 4. Results of day 5. [file 13075_2022_2728_MOESM4_ESM.pdf]

Additional file 4: Results of day 5

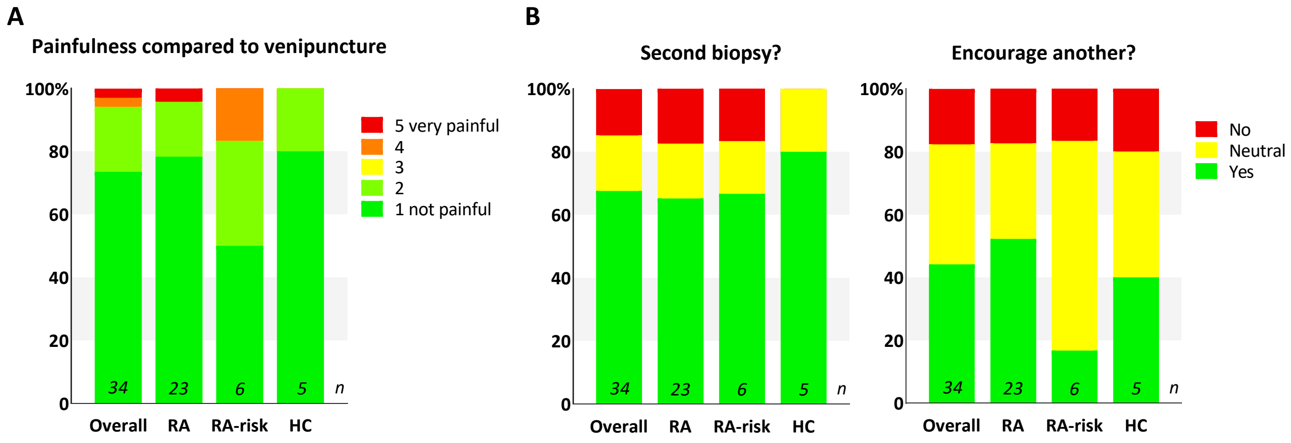

(A) Study participants scored the pain they experienced during the ultrasound-guided inguinal lymph node biopsy on a five point Likert scale from 1 `not painful´ to 5 ´very painful´ compared to venipuncture on day 5 after the biopsy procedure. The majority (94%) of responders scored either 1 or 2. (B) Study participants reported their willingness to undergo a second biopsy and to encourage someone else to participate in a similar study by choosing yes, no or neutral. The majority of all responders was willing to undergo a second biopsy (68%) and almost half of the participants was willing to encourage someone else (44%). Overall includes RA, RA-risk and HC individuals. RA, rheumatoid arthritis patients; RA-risk, individuals at risk for developing RA; HC, healthy controls.
