# Supplementary material for: Ultrasound-guided lymph node biopsy sampling to study the immunopathogenesis of rheumatoid arthritis: a well-tolerated valuable research tool
Source: Arthritis Res Ther. 2022 Feb 3;24:36. doi: 10.1186/s13075-022-02728-7 (PMC8812012; doi:10.1186/s13075-022-02728-7)
Supplement: Supplementary file 5 — Additional file 5. Self-reported complications at day 5. [file 13075_2022_2728_MOESM5_ESM.pdf]

Additional file 5: Self-reported complications at day 5

	Number of participants
Overall	17 / 34
- Hematoma	15
- Wound leakage	5
- Infection	0
- Bleeding	0
- Severe pain	3

Participants self-reported complications on the questionnaire and could choose multiple options.
