# Supplementary material for: Ultrasound-guided lymph node biopsy sampling to study the immunopathogenesis of rheumatoid arthritis: a well-tolerated valuable research tool
Source: Arthritis Res Ther. 2022 Feb 3;24:36. doi: 10.1186/s13075-022-02728-7 (PMC8812012; doi:10.1186/s13075-022-02728-7)
Supplement: Supplementary file 6 — Additional file 6. Explanatory statements that participants mentioned for their choice of yes/neutral/no considering a second ultrasound-guided lymph node biopsy or encouraging another to do so. [file 13075_2022_2728_MOESM6_ESM.pdf]

Additional file 6: Explanatory statements that participants mentioned for their choice of yes/neutral/no considering a second ultrasound-guided lymph node biopsy or encouraging another to do so

**Clarifying statement for second participation when answered yes, no or neutral**	**Clarifying statement for encouraging others to participate when answered yes, no or neutral**
**Yes**	**Yes**
The biopsy went 100% better than I expected beforehand<br>It was less painful than expected<br>Biopsy was not painful and it is important for research<br>If it aids medicine in finding a cure<br>A second biopsy would not be a problem<br>It went better than expected<br>The value for future patients outweighs the little pain and discomfort I endured<br>I have experienced no problems with the biopsy<br>(I would not do it to have fun,) but if necessary, no problem<br>I barely felt it and it is for a very good cause<br>If it is necessary for research<br>I would for research<br>I do not mind. It does not hurt, so I would undergo a second biopsy<br>If it is important for research<br>If it is necessary for further research<br>If it contributes to knowledge about RA, I would consider it<br>The procedure was not too painful and doable<br>The procedure went fine. I did experience some pain, but they immediately responded to that	I would explain that a biopsy seems way worse than it actually is<br>It is for a good cause<br>I have already encouraged another<br>We should help each other<br>Because others can benefit from it<br>Only if this person is mentally and physically up to undergo the procedure and feels the personal need to contribute to research<br>Because research is very important<br>To understand more about the disease. The general benefit is also very important.<br>As humans we should help each other<br>If the study is of great importance

**Neutral**	**Neutral**
It depends on the study goal and necessity of the study<br><br>It depends the goal of the study<br><br>Only if it is really needed<br><br>Currently, I experience too much pain from RA so two study participations is enough for me<br><br>I would do it to benefit research	Everyone should decide for themselves<br><br>I do not know anyone with RA , so I do not know who to ask<br><br>Depends on the goal of the study<br><br>I would not know who<br><br>Everyone should decide for themselves<br><br>I would describe my experience and leave the decision up to that person
**No**	**No**
Very painful<br><br>Once is enough	Very painful experience<br><br>Because the people I know, would not do it
