# Supplementary material for: Ultrasound-guided lymph node biopsy sampling to study the immunopathogenesis of rheumatoid arthritis: a well-tolerated valuable research tool
Source: Arthritis Res Ther. 2022 Feb 3;24:36. doi: 10.1186/s13075-022-02728-7 (PMC8812012; doi:10.1186/s13075-022-02728-7)
Supplement: Supplementary file 7 — Additional file 7. Questionnaire before ultrasound-guided lymph node biopsy (in English). [file 13075_2022_2728_MOESM7_ESM.pdf]

Additional file 7: Questionnaire before ultrasound-guided lymph node biopsy (in English)

Question 1.

Please state your sex:

Male	☐
Female	☐

Question 2.

Please state your age:

………………. years

The following questions concern your past experiences with biopsies or other medical procedures.

Question 3.

a. Have you ever undergone inguinal lymph node biopsy sampling under local anesthetic?

No	☐	If you answered 'no', please continue to question 4
Yes	☐	

b. If you answered 'yes' to question 3a: How many times have you undergone an inguinal lymph node biopsy under local anesthetic?

………………. times

c. When was the last time that you have undergone an inguinal lymph node biopsy under local anesthetic?

………/………. (month/year)

Question 4.

a. Have you ever undergone a procedure under local anesthetic?

No	☐	If you answered ‘no’, please continue to question 5
Yes	☐	

b. If you answered ‘yes’ to question 4a: How many times have you undergone a procedure under local anesthetic?

……………… times

c. If you answered ‘yes’ to question 4a: when was the last time that you have undergone a procedure under local anesthetic?

………/………. (month/year)

Question 5.

What are your reasons for participating in this study? Please check the appropriate box(es). You can select multiple boxes.

I have a family member or friend with rheumatoid arthritis (RA), so I know how RA can affect someone’s life and want to help.	☐
I would like to help decrease RA burden for future patients.	☐
I believe it is important to partake, if possible, in research projects to advance medicine in general.	☐
I heard about it from my general practitioner / rheumatologist and wanted to help	☐
Other, namely……………………………………………………………………………………………<br>………<br>………<br>………<br>………	☐

Question 6.
Please indicate with a vertical mark on the lines below, how well prepared you feel for the lymph node biopsy procedure with respect to:

a. understanding of the aim of the procedure and the background information

____

0 mm *100 mm*

I do not understand I completely understand

b. what to expect during the procedure

____

0 mm *100 mm*

I do not understand I completely understand

c. after-care

____

0 mm *100 mm*

I do not understand I completely understand

d. possible complications

____

0 mm *100 mm*

I do not understand I completely understand

Question 7.
Please indicate with a vertical mark on the lines below, how anxious you are about undergoing the procedure:

____

0 mm *100 mm*

I am not anxious I am very anxious

Question 8.
Please indicate with a vertical mark on the lines below, how much you dread to undergo the procedure:

____

0 mm *100 mm*

I do not dread the procedure I dread the procedure very much

Question 9.

Please tick the appropriate box(es) if you had to discontinue the following medication because of the lymph node biopsy procedure:

Anti-coagulants	☐
Pain medication	☐

Please elaborate below, if you have any remarks about this questionnaire.

……
……
……
……
……
……
……
……
……
……
……
……
……
……

Thank you for your cooperation!
