# Supplementary material for: Ultrasound-guided lymph node biopsy sampling to study the immunopathogenesis of rheumatoid arthritis: a well-tolerated valuable research tool
Source: Arthritis Res Ther. 2022 Feb 3;24:36. doi: 10.1186/s13075-022-02728-7 (PMC8812012; doi:10.1186/s13075-022-02728-7)
Supplement: Supplementary file 8 — Additional file 8. Questionnaire 1 and 5 days after ultrasound-guided lymph node biopsy (in English). [file 13075_2022_2728_MOESM8_ESM.pdf]

Additional file 8: Questionnaire 1 and 5 days after the ultrasound-guided lymph node biopsy (in English)

<u>Question 1.</u>
Compared to venipuncture, I find the lymph node biopsy procedure (please check the appropriate box):

Not painful Very painful

☐	☐	☐	☐	☐
1	2	3	4	5

<u>Question 2.</u>

a. Have you experienced any complications following the procedure?

No	☐	If you answered 'no', please continue to question 3
Yes	☐	

b. If you answered 'yes' to question 2a, please mark which complication(s) you experienced:

1. Hematoma:

No	☐
Yes	☐

2. Wound leakage:

No	☐
Yes	☐

3. Bleeding:

No	☐
Yes	☐

4. Inflammation:

No	☐
Yes	☐

5. Severe pain:

No	☐
Yes	☐

6. Other complications, not mentioned before:

No	☐
Yes, namely………………………………………………………………………………………………… ………	☐

<u>Question 3.</u>

a. In hindsight, which aspects of the lymph node biopsy procedure could have been better explained?

None, I am content with the information that was given	☐

or:

More detailed explanation of the procedure itself	☐
The anesthetics procedure	☐
After-care	☐
Possible complications	☐
Other, namely ……………………………………………………………………………………… ……… ………	☐

b. If you ticked any of the boxes above, please explain which information you feel should have been given to prepare you for the procedure:

……… ……… ……… ……… ………

<u>Question 4.</u>

In hindsight, do you feel anything could have improved your experience during the lymph node biopsy procedure?

No	☐
Step-by-step explanation of what happens during the procedure	☐
Distraction, for instance listening to music or jokes.	☐
Other, namely……………………………………………………………………………………… ……… ………	☐

Question 5.

a. Would you consider undergoing another lymph node biopsy procedure?

No	☐
Yes	☐
Neutral	☐

b. Please explain your choice:

……<br>……<br>……	☐

Question 6.

a. Would you encourage someone else to partake in a research study that involves an inguinal lymph node biopsy procedure?

No	☐
Yes	☐
Neutral	☐

b. Please explain your choice:

……<br>……<br>……	☐

Question 7.

Would you like to be informed about the study results after the study has finished?

No	☐
Yes	☐

Please elaborate below, if you have any remarks about this questionnaire.

……
……
……
……

Regarding the questionnaires before and after the procedure: please let us know if any of the questions were unclear and how we might improve these questions.

……
……
……
……

Thank you for your cooperation!
